# The Effect of Neoglandin on the Activity of N-Acetyl-β-D-Hexosaminidase in the Serum and Urine of Alcohol-Dependent Men

**DOI:** 10.3390/ijerph20043413

**Published:** 2023-02-15

**Authors:** Sławomir Dariusz Szajda, Jacek Dobryniewski, Alina Minarowska, Jadwiga Snarska, Napoleon Waszkiewicz, Krzysztof Zwierz

**Affiliations:** 1 Department of Emergency Medical Service, University of Warmia and Mazury in Olsztyn, 10-561 Olsztyn, Poland; 2 DJV Sp. z.o.o., 15-660 Bialystok, Poland; 3 Department of Pulmonology Collegium Medicum, University of Warmia and Mazury in Olsztyn, 10-357 Olsztyn, Poland; 4 Department of Surgery, Collegium Medicum University of Warmia and Mazury in Olsztyn, 10-228 Olsztyn, Poland; 5 Department of Psychiatry, Medical University of Bialystok, 15-272 Bialystok, Poland; 6 Academy of Applied Sciences in Lomza, 18-400 Lomza, Poland

**Keywords:** alcohol addiction, neoglandin, N-acetyl-β-D-hexosaminidase (HEX), serum, urine

## Abstract

Dietary supplementation of gamma-linolenic acid (GLA) in the form of a commercial drug neoglandin (containing GLA and vitamin E), in people following alcohol abuse allows bypassing of the ineffective delta-6-desaturase system involved in the transformation of linoleic acid into GLA. Determination of the activity of N-acetyl-β-D-hexosaminidase (HEX) in the serum and urine reflects neoglandin action on the catabolism of glycoconjugates and the functioning of liver and kidneys in people following alcohol abuse. Material and methods: The serum and urine were collected from men with alcohol dependence, treated (*n* = 31, age 33.16 ± 9.72 years) and not treated (*n* = 50, age 35.46 ± 11.37 years) with neoglandin. HEX activity were assayed in the supernatants by the colorimetric method, with the p-nitrophenyl derivative of sugar as substrate. Results: Our study on alcoholic men not treated with neoglandin indicates a significantly higher concentration of the serum and urinary HEX activity (nKat/L) on day 1 compared to days 7, 10, 14 and 30 (*p* < 0.001). For days 14 and 30 (*p* < 0.01), the urinary HEX activity was expressed in μKat/kgCr. No significant differences were observed in the activity of serum (nKat/L) and urinary (nKat/L and μKat/kgCr) HEX in alcoholics during treatment with neoglandin compared to day 1 of neoglandin treatment. We found significantly different (*p* < 0.05) concentration of HEX activity (nKat/L) in serum of alcohol-dependent men treated with neoglandin compared to those not taking neoglandin on days 7, 10, 14 and 30 of treatment. The urinary concentration of HEX activity (nKat/L) on days 1, 4, 10 and 30 and HEX activity in μKat/kgCr on days 1, 4 and 7 it was significantly higher (*p* < 0.05) during the treatment of alcohol-dependence without the use of neoglandin as compared to alcoholics treated with neoglandin. We found a positive correlation between the amount of alcohol consumed and the urinary activity of HEX in the early phase after alcohol withdrawal and a lack of correlation between the HEX activity in serum and urine of alcohol-dependent men not treated with neoglandin. Conclusions: Neoglandin supplementation in alcoholic men significantly slows down the catabolism of glycoconjugates, thus reducing the effects of ethanol poisoning that are harmful to the kidneys. Neoglandin reduces the harmful effects of ethanol poisoning more on the kidneys than on the liver. The activity of HEX in the serum may be used in monitoring the treatment of alcoholism and whether alcohol reuse occurred during the therapy. In the early stages of alcohol withdrawal, urinary HEX activity can be used as a marker of the amount of alcohol consumed during previous alcohol abuse.

## 1. Introduction

Alcohol and its metabolites interfere with the functioning of many human body organs, causing several diseases. Alcohol provides the human body with empty calories, disrupts the energy balance, and leads to nutritional deficiencies. The oxidation of ethanol in the liver by alcohol dehydrogenase results in the formation of toxic acetaldehyde with the production of NADH and the reduction of NAD/NADH. These processes lead to excessive fat accumulation in the liver; hence, hepatic steatosis is the first symptom of alcohol abuse [1,2,3].

In the chronic alcohol abuse, the second hepatic pathway of alcohol metabolism (MEOS) gains the upper hand, where cytochrome P 450 plays a key role. This pathway of metabolism, in addition to the additional potentiation of the conversion of alcohol to acetaldehyde, causes oxygen deficiency leading to cell and tissue damage and energy deficits, impaired fat and fatty acid metabolism and oxidative stress [4].

Acetaldehyde, on the other hand, leads to protein damage, impaired DNA repair and excessive collagen production [5].

There is an association between ethyl alcohol abuse and metabolic disorders of essential fatty acids (EFA). Chronic alcohol consumption provides the dependent person with about 50% of his or her caloric demand, which changes the metabolism of EFA [6]. Ingested ethanol inhibits the activity of delta-6-desaturase and delta-5-desaturase, which are key enzymes involved in the transformation of linoleic acid (LA) to longer chains of the unsaturated fatty acids gamma-linolenic acid (GLA) and dihomo-gamma-linolenic acid (DGLA), as well as arachidonic acid (AA) [6,7,8,9].

The delta-6-desaturase block of the ingested alcohol, causes a deficiency of essential fatty acids, resulting from blocking the conversion of essential unsaturated fatty acids (EUFA) at the level of transformation of linoleic acid to GLA and DGLA [10]. GLA supplementation with neoglandin (containing vitamin E) allows EUFA to bypass the ineffective delta-6-desaturase system while providing an increased amount of DGLA precursor. This produces AA and other important metabolites of EFA [10].

Current options for treating damage caused by alcohol and its metabolites in humans are very limited. The positive effect of polyunsaturated fatty acids and vitamin E on liver damage caused by alcohol and free radicals has been evaluated [11,12,13].

There are also reports that GLA has a positive effect on the treatment of atopic dermatitis, age-related diseases, diabetic neuropathy, and alcohol dependence [14].

Currently, self-assessment questionnaires and biological markers are used in the diagnosis of alcoholism. The limitation of self-assessment questionnaires is due to non-disclosure of alcoholism by the patient in clinical settings and additional stress during the study [15]. Alcohol abuse biological markers include gamma-glutamyl transferase (GGT), aspartate aminotransferase (AST), alanine aminotransferase (ALT), mean corpuscular volume (MCV), and the low-carbohydrate isoform of transferrin (CDT), or desialated transferrin. The concentrations of these biological markers in blood serum correlates with the intensity of alcohol consumption. However, biological markers are characterized by too low sensitivity and specificity to be treated as reliable tests. Biological markers are useful for general clinical evaluation and may suggest the existence of alcoholism [16].

In addition to these markers of alcoholism, changes in the activity of N-acetyl-β-hexosaminidase (HEX) in serum, urine, and saliva [17,18,19] should be considered as an indicator. HEX is the most active of lysosomal exoglycosidases, hydrolyzing N-acetylglucosamine and N-acetyl galactosamine residues from the non-reducing ends of the oligosaccharide chains of glycoproteins, glycolipids, and proteoglycans, collectively referred to as glyconjugates [20]. HEX activity in serum and urine correlates with the duration and amount of ethanol consumption and is a sensitive indicator of ethyl alcohol abuse [19].

The aim of our study was to assess the effect of neoglandin (borage oil, GLA, and vitamin E) treatment on the activity of HEX in the serum and urine of patients hospitalized due to long-term alcoholism.

## 2. Materials and Methods

The study group consisted of 31 men aged 20–56 years (mean 33.16 ± 9.72 years) hospitalized after long-lasting alcoholism at the Detoxification Wards and the Alcohol Dependence Therapy Unit at SPPZOZ in Choroszcz, Poland. The men received threeneoglandin capsules (369 mg borage seed oil, polyunsaturated essential fatty acids 203 mg, gamma-linolenic acid (GLA) 74 mg, vitamin E 1.0 mg) twice daily in addition to the standard treatment starting from the first day of their hospitalization. The patients of the study met the alcohol dependence criteria by ICD-10 classification and were admitted to the hospital directly after alcohol abuse for detoxification and further treatment of alcohol dependence. The length of time when alcohol was abused ranged from 4 to 90 days (average 23.74 ±17.60 days). The declared daily intake of alcohol calculated as 100% ethanol was from 80 to 400 g (average 219.68 ± 87.65 g).

The control group consisted of patients hospitalized in the same departments and for the same indications as in the study group. Patients of the control group underwent only standard treatment, without further neoglandin treatment. The control group consisted of 50 men aged 20–67 years (mean 35.46 ± 11.37 years). The control group patients were admitted to the ward after a recent excessive alcohol consumption lasting from 4 to 180 days (mean 28.58 ± 33.27 days). The amount of alcohol consumed calculated per 100% ethanol was 80 to 800 g (average 317.0 ± 168.71 g).

Patients disqualified from the study included those with non-ethanol addiction disorders that may be associated with increased NAG activity in the urine e.g., severe non-alcoholic liver disease, myocardial infarction, hypertension, diabetes, sepsis, kidney diseases, urinary tract infections, and thyrotoxicosis. Serum and urine samples were collected on days 1, 4, 7, 10, 14 and 30 of abstinence from alcohol. During hospitalization, patients were intravenously administered 5% glucose, multi-electrolyte fluid, and 0.9% NaCl. They were also intramuscularly or orally administered haloperidol (up to 20 mg/day), diazepam (up to 50 mg/day), and B-group vitamins.

Blood was collected on an empty stomach. The collection was made from the elbow vein at a fixed time—in the morning. Urine samples were collected on the first morning of hospitalization from the middle stream. Blood (after clotting) and urine were centrifuged for 20 min at 3.000 rpm, divided into aliquots, and stored in Eppendorf plastic tubes at −86 °C until the assays were made.

HEX activity in the serum and urine was determined by the Chatterjee et al. method [21] modified by Zwierz et al. [22]. To 50 µL of the serum and urine samples to be tested was added 200 µL of 0.1 M phosphate-citrate buffer at pH 4.7, and 150 µL of 20 mM substrate solution of p-Nitrophenyl-N-acetyl-β-D-glucosaminide (Sigma, St. Louis, MO, USA), and then incubated for 60 min in a warmbath at 37 °C. The reaction was stopped by adding 1 mL of 0.2 M borate buffer, at pH 9.8, to the incubated solution. The activity HEX, corresponding to the amounts of released p-nitrophenol, were measured at 410 nm, using a Specol spectrophotometer. The amount of p-nitrophenol released in the reaction was read from the calibration plot. Creatinine concentration was determined by the kinetic method [23] using the Mascott Lisa Plus analyzer (Hycel Diagnostic, Massy, France).

The study was carried out with the approval of the Bioethical Commission of the Medical University of Bialystok.

In statistical analysis, the normality of distribution was verified by Kolmogorov–Smirnov tests with the Lilliefors correction and the Shapiro-Wilk test. A normal distribution of the analyzed quantitative variables was not produced. Because the quantitative variables did not produce a normal distribution, the non-parametric Mann–Whitney U test was used for the two groups. For the dependent variables, the Friedman ANOVA test was used with the adjusted post hoc Conover test. The Spearman rank correlation coefficient was also determined. Statistically significant results were found at *p* < 0.05. The Statistica 10.0 package from StatSoft and PASW Statistics 17.0 from Predictive Solutions were used for the calculations.

## 3. Results

**Figure 1:**
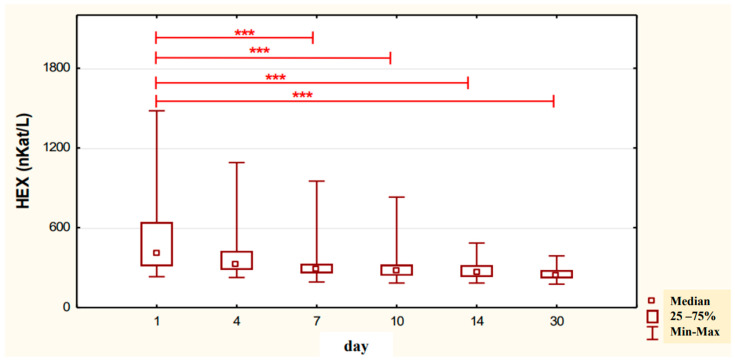
Variability over time in the concentration of serum HEX activity (nKat/L) of control group patients addicted to alcohol but not treated with neoglandin (K); *** *p* < 0.001.

**Figure 2:**
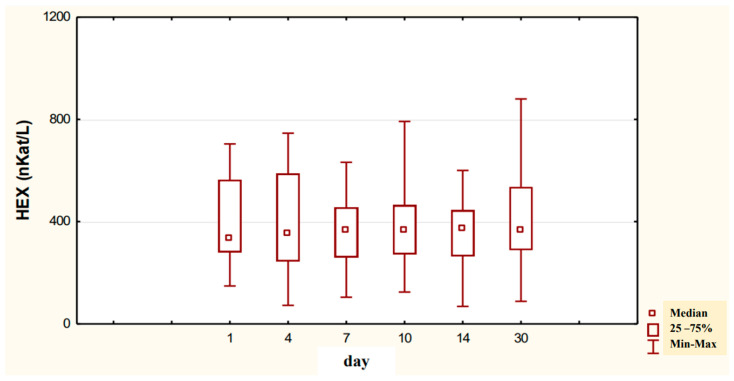
Variability over time in the concentration of serum HEX activity (nKat/L) of alcohol-dependent patients treated with neoglandin (N).

**Figure 3:**
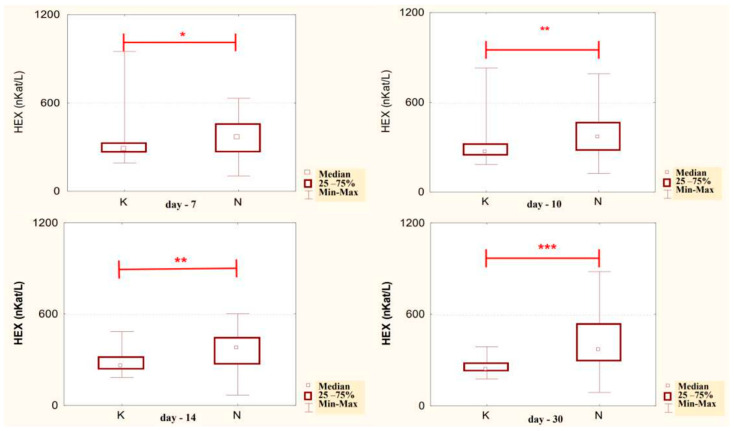
Serum HEX activity concentration (nKat/L) in patients addicted to alcohol and treated with neoglandin (N) compared to the control group patients (K) who were addicted to alcohol but not treated with neoglandin; * *p* < 0.05, ** *p* < 0.01, *** *p* < 0.001.

The conducted studies suggest that in the serum of male alcoholics not treated with neoglandin, the concentration of HEX activity (nKat/L) was significantly higher (*p* < 0.001) on day 1 compared to days 7, 10, 14 and 30 of treatment (Figure 1). In the serum of neoglandin-treated alcoholics, there were no significant differences in HEX activity concentration (nKat/L) on days 4, 7, 10, 14, 30, compared to day 1 of treatment for alcohol dependence with neoglandin (Figure 2). Significantly higher (*p* < 0.05) concentration of HEX activity (nKat/L) was found in the serum of alcohol-dependent men treated with neoglandin compared to those who did not take neoglandin on days 7, 10, 14 and 30 of treatment (Table 1, Figure 3).

**Figure 4:**
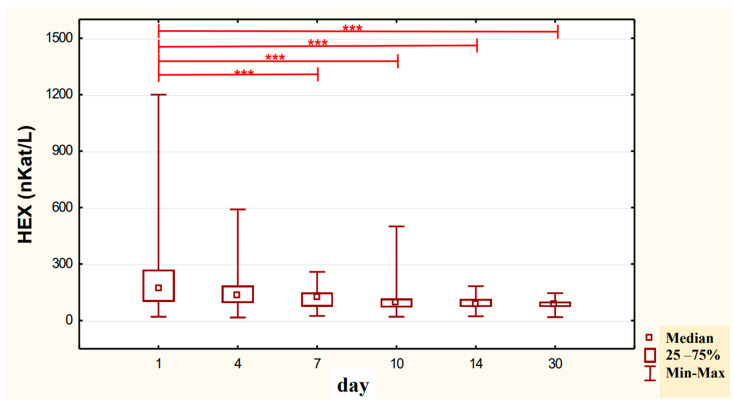
Variability over time in the concentration of urinary HEX activity (nKat/L) of control group patients addicted to alcohol but not treated with neoglandin (K); *** *p* < 0.001.

**Figure 5:**
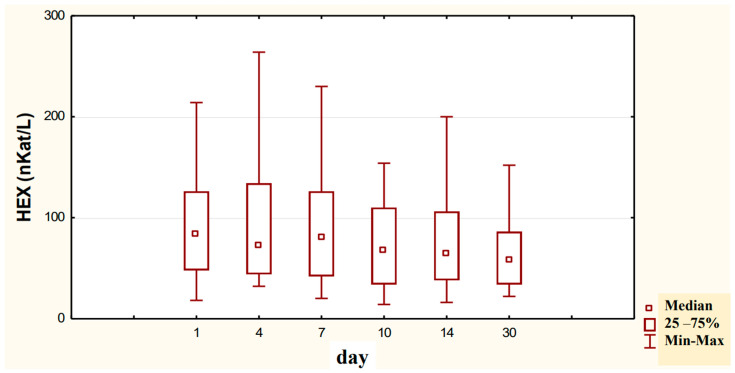
Variability over time in the concentration of urinary HEX activity (nKat/L) of alcohol-dependent patients treated with neoglandin (N).

**Figure 6:**
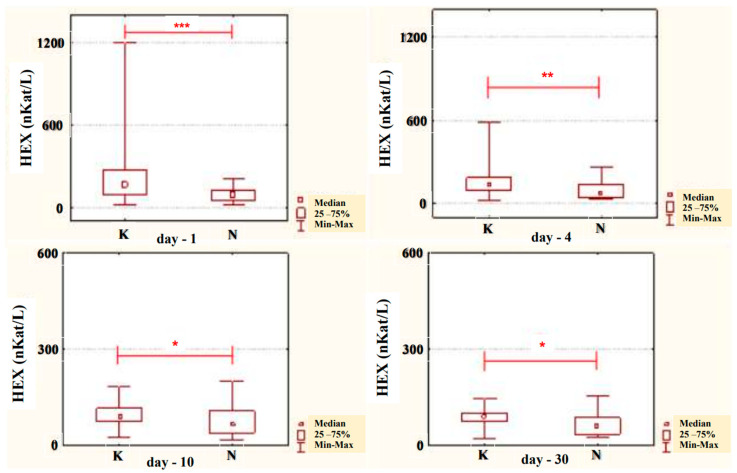
Urinary HEX activity concentration (nKat/L) in patients addicted to alcohol and treated with neoglandin (N) compared to the control group patients (K) who were addicted to alcohol but not treated with neoglandin; * *p* < 0.05, ** *p* < 0.01, *** *p* < 0.001.

**Figure 7:**
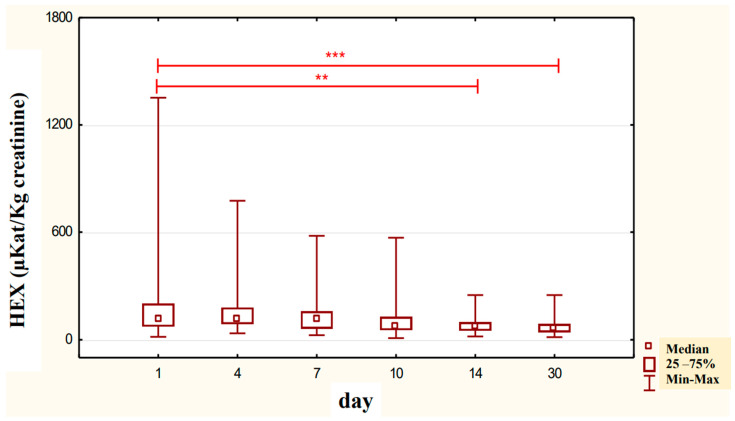
Urinary HEX specific activity (μKat/kg creatinine) of control group patients (K), in subjects who were addicted to alcohol but not treated with neoglandin; ** *p* < 0.01, *** *p* < 0.001.

**Figure 8:**
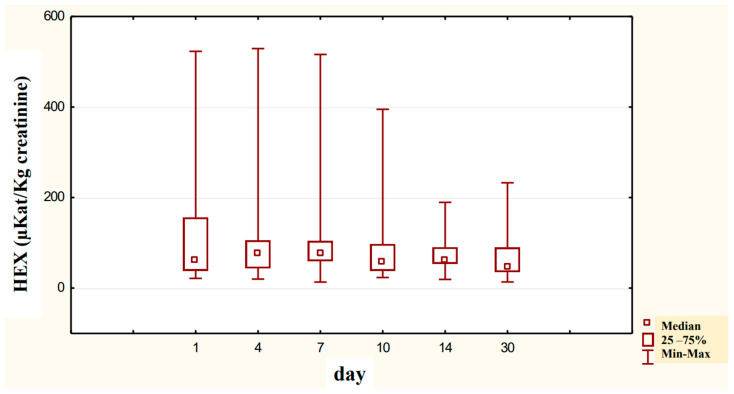
Variability over time of specific urinary HEX activity (μKat/kg creatinine) of alcohol-dependent patients treated with neoglandin (N).

**Figure 9:**
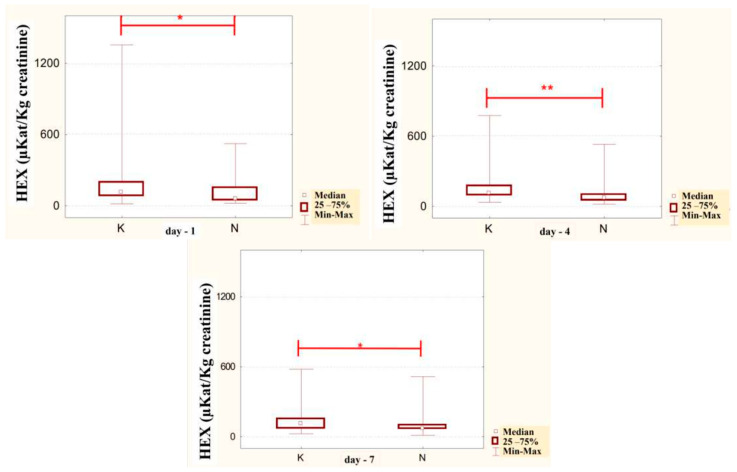
Specific urinary HEX activity (μKat/kg creatinine) in patients addicted to alcohol and treated with neoglandin (N) compared to the control group patients (K) who were addicted to alcohol but not treated with neoglandin; * *p* < 0.05; ** *p* < 0.01.

In the urine of alcoholic men not taking neoglandin, the concentration of the HEX activity (nKat/L) was significantly higher (*p <* 0.001) on day 1 compared to days 7, 10, 14 and 30 of treatment (Figure 4). Found no significant differences in HEX activity concentration (nKat/L) in the urine on days 4, 7, 10, 14, 30, compared to day 1 of treatment for alcohol dependence with neoglandin (Figure 5). Significantly higher (*p <* 0.05) concentration of HEX activity (nKat/L) were found in the urine of alcohol-dependent men not treated with neoglandin compared to those taking neoglandin on days 1, 4, 10 and 30 of treatment (Table 2 and Figure 6). In the urine of alcoholic men not taking neoglandin, the concentration of HEX activity calculated per kg creatinine (μKat/kgCr) was significantly higher (*p <* 0.01) on day 1 compared to day 14 and 30 of treatment (Figure 7). No significant differences in the concentration of HEX activity per kg of creatinine (μKat/kgCr) in the urine were found on days 4, 7, 10, 14, 30 compared to day 1 of treatment for alcohol-dependence with neoglandin (Figure 8). A significantly higher (*p <* 0.05) concentration of HEX activity after the conversion to kg of creatinine (μKat/kgCr) was found in the urine of men with alcohol-dependence without neoglandin treatment, as compared to those taking neoglandin on days 1, 4, and 7 of treatment (Table 3 and Figure 9).

There appears to be a positive correlation between the amount of alcohol consumed and the activity of HEX in the urine in the early phase of alcohol withdrawal, which suggests the possibility of using HEX as a marker of the amount of alcohol consumed (Table 4).

## 4. Discussion

HEX activity has been demonstrated in many tissues and organs: kidney, spleen, liver, gastric and intestinal mucosa, cerebral cortex, skin fibroblasts, placenta, and cancerous tissues. This enzyme has also been detected in biological fluids such as serum, urine, cerebrospinal fluid, and synovial fluid [24].

Ethanol and its metabolites: acetic aldehyde, free radicals, and fatty acid ethyl esters, as well as the ethanol-water competition mechanism, disturb the processes of glycoconjugate metabolism (biosynthesis, modification, transport, secretion, elimination and catabolism) [25]. Since most ingested alcohol and most plasma glycoproteins are metabolized in the liver, the two processes interfere with each other, resulting in impaired metabolism of the glycoconjugates.

Previous studies indicated the increasing activity of HEX in alcoholics. The average HEX levels were twice as high in alcoholics after drinking alcohol compared to the so-called social drinkers and total abstainers. The sensitivity of HEX in detecting heavy drinking, defined as 60 g of pure ethanol per day, was 85.7% compared to 47.6% for GGT. Positive correlations between HEX and AST (r = 0.74, *p <* 0.0001) and ALT (r = 0.41, *p <* 0.05) [24] indicate that elevated HEX levels may reflect early liver damage. Plasma HEX seems to be a sensitive biological marker of heavy drinking, better reflecting the amount of ethanol ingested than GGT [24].

Our study shows that in the serum of male alcoholics not treated with neoglandin the concentration of HEX activity (nKat/L) was significantly higher (*p* < 0.001) on day 1 compared to days 7, 10, 14 and 30 of treatment (Figure 1). In the group of men not treated with neoglandin, the concentration of HEX activity decreased significantly over time, and on day 30 of treatment, it was more than one and a half times lower compared to day 1 of treatment, reaching values referred to as normal [24,26] (Table 1).

The obtained results are consistent with the studies of other authors [24,26,27], in which they showed that in people addicted to alcohol, after several days of drinking, the activity of plasma HEX on the first day of abstinence was significantly higher than the activity of HEX determined in the group of people drinking socially or abstinent. The study of the concentration of HEX activity in the serum of alcohol-dependent men treated with neoglandin suggests no significant differences in HEX activity between individual days of blood collection (Figure 2). In the serum of men taking neoglandin, we did not observe the expected faster decrease in HEX activity compared to men not taking neoglandin. On the other hand, increased HEX activity in the serum of men addicted to alcohol was found up to 30 days of neoglandin therapy, which suggests that neoglandin as a whole or one of its components supports increased catabolism of glycoconjugates in lysosomes and contributes to high HEX activity.

Comparing the concentrations of HEX activity in the serum of men addicted to alcohol not treated with and treated with neoglandin, significantly lower HEX activity was found on days: 7 (*p* < 0.05), 10 (*p* < 0.01), 14 (*p* < 0.01) and 30 (*p* < 0.001) in untreated versus neoglandin-treated men (Table 1 and Figure 3). The difference in the concentration of HEX activity was on average about 30% in favor of the group receiving the neoglandin preparation. These results suggest that the neoglandin preparation stimulates the lysosomal catabolism of HEX containing glycoconjugates.

It seems that the reason for the increased activity of HEX in the serum of men addicted to alcohol and treated with neoglandin may be borage oil, which increases the level of DGLA, causing an increase in the level of prostaglandins of the 1 series, e.g., PGE 1, which, like other prostaglandins, is involved in the inflammatory process, causing redness, swelling, pain, increased temperature, and partial or complete loss of function of a given organ. However, the effect of PGE 1 on segmented nucleated leukocytes is mainly inhibitory [28]. PGE1 increases the concentration of intracellular cyclic AMP (cAMP), which in turn reduces the release of lysosomal enzymes, the chemotaxis of polymorphonuclear leukocytes and the margination and adhesion of leukocytes in blood vessels. It is also believed that PGE1 inhibits the activity of lymphocytes [29]. Exogenous PGE1 inhibits both lymphocyte function in vitro and lymphocyte-mediated reactions in vivo. There is an opinion that PGE1 initially promotes the formation of the inflammatory process to inhibit inflammation at a later stage. Bonta and Pernham [30] showed that PGE1 administered to rats fed a diet devoid of EFA (producing meagre amounts of prostaglandins) induced a pro-inflammatory effect during the first ten days of the experiment through dilatation and increased permeability of vessels, which resulted in increased migration of granulocytes, which effect was gradually replaced by the opposite effect—anti-inflammatory effect induced by cAMP.

The obtained results may suggest that neoglandin as a whole or some of its components, such as, e.g., borage oil, stimulates the lysosomal catabolism of glycoconjugates through its pro-inflammatory effect. In the next series of experiments, it is worth examining the effect of the individual components of the neoglandin preparation on the activity of serum HEX and other indicators of chronic consumption of excessive amounts of ethanol.

In order to assess whether neoglandin has a positive or negative effect on the metabolism of glycoconjugates, it would be necessary to study not only the effect of neoglandin on the breakdown of glycoconjugates, e.g., by determining HEX activity, but also the effect of neoglandin on the rate of biosynthesis of glycoconjugates.

Determination of urinary enzyme activities is a sensitive and non-invasive method for assessing renal tubular function. Out of more than 50 enzymes produced in the epithelium of the proximal renal tubules and secreted to urine, only several have found application in medical diagnostics [31].

HEX is a recognized, useful, low-cost marker for assessing proximal renal tubular function. Increased HEX secretion into the urine is observed in acute and chronic glomerulonephritis and after poisoning with heavy metal salts. Determining HEX in urine has found application in monitoring of drug nephrotoxicity and renal status after transplantation [31].

Proteins with a molecular weight greater than 68 kD [32] do not pass through renal glomeruli, and HEX is not filtered through a properly functioning renal tubular membrane filter. Nevertheless, low HEX activity was observed in the urine of healthy people, which indicates that the breakdown of the renal cells and damage to the membranes results in the release of HEX into the urine. The reason for the presence of HEX in physiological urine may be that under normal cell metabolism, the lysosomal enzymes can be released out of the cell and into the urine [33].

After analysis of the concentration of HEX activity (nKat/L) in the urine of alcoholic men not treated with neoglandin, we found a significant decrease in HEX activity (*p* < 0.001) in days 7, 10, 14, and 30 of treatment as compared to day 1 of treatment (Figure 4). The study shows that the concentration of HEX (nKat/L) activity in the urine of alcoholic men supplemented with neoglandin decreased over time. On day 30 of the neoglandin treatment, the concentration of urinary HEX activity (Table 2 and Figure 5) was similar to the concentration of urinary HEX activity observed in healthy people [34].

Our study on the concentration of the urinary HEX (nKat/L) activity in alcoholic men treated with neoglandin indicates no significant changes in HEX activity during treatment with neoglandin (Figure 5).

Upon comparing the levels of HEX activity in the urine of alcoholic men, we observed significantly lower HEX activity on days 1 (*p* < 0.001), 4 (*p* < 0.01), 10 (*p* < 0.05) and 30 (*p* < 0.05), in men treated versus those not treated with neoglandin (Table 2 and Figure 6).

Positive results of neoglandin treatment on HEX activity in the urine of alcoholic men have been observed since the beginning of neoglandin treatment for alcohol-dependence (Table 2 and Figure 5). The difference in concentration of HEX (nKat/L) activity between the alcoholic men treated and not treated with neoglandin was, on average, 30% in favor of men taking neoglandin. In this group, the HEX (nKat/L) activity in urine was lower than in the urine of men not treated with neoglandin. Our results suggest that the neoglandin treatment positively impacts the kidneys, reducing the harmful effects of the consumed ethanol’s nephrotoxic action.

It has been reported that urinary creatinine concentration depends directly on muscle mass and magnitude of renal excretion and significantly decreases during fasting and acute and chronic renal failure [35]. It is accepted that the best way to eliminate the effect of diuresis on HEX concentration and HEX activity in urine is to compare HEX activity to urine creatinine. Calculation of urinary HEX activity on creatinine excludes the influence of individual variations on the urinary activity of HEX [36]. Our study found that 30-day neoglandin therapy did not significantly affect renal excretion of creatinine compared to alcohol-dependent males who were not treated with neoglandin. Therefore, the results of urinary HEX activity calculated per 1 kg of creatinine (Table 3 and Figure 7, Figure 8 and Figure 9) seemed to be valuable, as it indicated a significant decrease in urinary HEX activity on day 14 (*p* < 0.01) and 30 (*p* < 0.001) of treatment of alcoholic men not treated with neoglandin as compared to day 1 of treatment (Figure 7).

The concentration of urinary HEX activity calculated per 1 kg of creatinine (μKat/kgCr) indicated no significant differences in urinary HEX activity on particular days of neoglandin treatment as compared to day 1 of neoglandin treatment (Figure 8). The comparison of urinary HEX activity calculated per 1 kg of creatinine (μKat/kgCr) indicated a significantly higher urinary HEX activity for men not treated with neoglandin compared to those treated with neoglandin on days 1 (*p* < 0.05), 4 (*p* < 0. 01) and 7 (*p* < 0.05), and the absence of significant growth of urinary HEX activity on days 10, 14 and 30 (Table 3 and Figure 9). The reduction in urinary concentration of HEX activity (nKat/L) and urinary HEX activity calculated per 1 kg of creatinine (μKat/kgCr) in the following days, as compared to the first day of neoglandin treatment (Table 2 and Table 3), may indicate that the toxic effects of alcohol do not cause permanent kidney damage in alcoholic men. In our study, it seems significant that the HEX activity values in the urine of men treated with neoglandin remained at normal levels. This may suggest that neoglandin supplementation has a positive effect on the kidneys.

Based on our urinary HEX study, it can be concluded that neoglandin reduces the harmful effects of ethanol poisoning on the kidneys because neoglandin supplementation significantly reduces the urinary HEX activity of alcoholic men compared to alcoholic men not treated with neoglandin.

A positive correlation between the amount of alcohol consumed by alcoholic men and the urinary HEX activity in the early phase of alcohol withdrawal suggests that HEX could serve as a marker for measuring of alcohol consumption (Table 4).

The lack of correlation between the concentration of HEX activity in serum and urine and the concentration of HEX activity in serum and the HEX activity calculated per 1 kg of creatinine in the urine suggests that the HEX activity in the urine of alcohol-dependent men not treated with neoglandin does not depend on the HEX activity in the serum, which may be due to the toxic effects of alcohol on the kidneys.

## 5. Conclusions

1. Long-term alcohol abuse and diet supplementation with neoglandin slow down the catabolism of glycoconjugates.
2. Neoglandin reduces the harmful effects of ethanol poisoning more on the kidneys than on the liver.
3. The concentration of HEX activity in the serum may be used in monitoring the treatment of alcoholism and whether alcohol reuse occurred during the therapy.
4. The concentration of urinary HEX activity of alcoholic men in the early phase of alcohol withdrawal may be used as a marker for measuring alcohol consumption.
5. Determination of the concentration of serum and urinary HEX activity is a cheap and easy method for diagnosing alcohol addiction.

## Figures and Tables

**Table 1:** The concentration of serum HEX activity (nKat/L) in alcohol-dependent patients treated with neoglandin (N1–N30) and the control group not treated with neoglandin (K1–K30). Blood samples were collected on days 1, 4, 7, 10, 14 and 30 of hospitalization.

Group Symbols	*N*	Average $\bar{x}$	Median Me	Standard Deviation ±SD	Probability of Random Differences *P*
**K1**	50	498.780	402.000	261.858	0.065
**N1**	30	396.233	332.000	162.800	
**K4**	49	380.327	322.000	163.750	0.957
**N4**	31	390.645	352.000	178.634	
**K7**	48	322.792	284.000	131.578	**0.022**
**N7**	31	367.742	364.000	124.861	
**K10**	41	297.854	272.000	109.649	**0.006**
**N10**	29	385.793	368.000	157.439	
**K14**	44	283.182	260.000	77.260	**0.008**
**N14**	27	355.704	376.000	130.419	
**K30**	38	254.263	243.000	56.552	**0.000**
**N30**	23	404.348	368.000	179.336	

**Table 2:** The concentration of urinary HEX activity (nKat/L) in alcohol-dependent patients treated with neoglandin (N1–N30) and the control group not treated with neoglandin (K1–K30). Urine samples were collected on days 1, 4, 7, 10, 14 and 30 of hospitalization.

Group Symbols	*N*	Average $\bar{x}$	Median Me	Standard Deviation ±SD	Probability of Random Differences *P*
**K1**	47	219.574	170.000	191.916	**0.000**
**N1**	29	90.759	84.000	59.319	
**K4**	48	158.000	132.000	113.154	**0.001**
**N4**	30	89.933	73.000	54.184	
**K7**	46	115.413	120.000	56.658	0.056
**N7**	27	90.593	80.000	57.710	
**K10**	40	103.550	94.000	75.841	**0.013**
**N10**	27	69.111	68.000	38.239	
**K14**	42	88.857	89.000	35.139	0.141
**N14**	25	76.560	64.000	45.671	
**K30**	37	86.595	88.000	29.245	**0.013**
**N30**	21	65.619	58.000	37.649	

**Table 3:** The urinary activity of HEX (μKat/kg creatinine) in those addicted to alcohol treated with neoglandin (N1-N30) and the control group which was not treated with neoglandin (K1-K30). Urine samples were collected on days 1, 4, 7, 10, 14 and 30.

Group Symbols	*N*	Average $\bar{x}$	Median Me	Standard Deviation ±SD	Probability of Random Differences *P*
**K1**	42	204.791	115.258	242.785	**0.015**
**N1**	28	115.632	62.164	122.059	
**K4**	43	158.100	114.286	140.330	**0.003**
**N4**	29	108.138	76.316	116.101	
**K7**	43	134.585	113.043	102.799	**0.048**
**N7**	26	100.613	74.602	96.720	
**K10**	40	109.301	73.190	106.209	0.162
**N10**	27	85.565	58.333	83.968	
**K14**	39	84.134	74.468	49.898	0.782
**N14**	23	82.304	62.745	48.212	
**K30**	37	74.150	67.114	46.320	0.389
**N30**	20	70.554	47.586	54.996	

**Table 4:** Correlations of variables in the urine of the control group.

CONTROL GROUP			
	*n*	R	*P*
**URINE**			
Positive correlation			
ALT d4/GGTP d4	30	0.7	0.000
ALT d10/GGTP d10	30	0.8	0.000
hex d1/creatinined1	42	0.4	0.011
hex d4/creatinined4	43	0.5	0.001
hex d7/creatinined7	43	0.3	0.024
hex d14/creatinined14	39	0.5	0.004
hex d30/creatinined30	37	0.5	0.004
alcohol a day/hex d4	48	0.4	0.009
alcohol a day/hex d10	40	0.3	0.031
alcohol a day_kg/hex d4	48	0.4	0.007
alc. per month/hex d4	48	0.4	0.002
alc. per month/hex d7	46	0.4	0.008
alc. per month/hex d10	40	0.5	0.002
alc. per month/hex d14	42	0.4	0.007
age/hex d1	47	0.4	0.007
age/hexcw30	37	0.3	0.038
age/hex_kg_creat. d7	43	0.3	0.034
alc. per month/creat. d4	43	0.3	0.029
alcohol a day/creat. d4	43	0.3	0.036
alcohol a day_kg/creat. d4	43	0.3	0.048
Negative correlations			
Hexcw30/cerat. d30	37	−0.6	0.000

## Data Availability

The data supporting reported results can be obtained from the corresponding author on request.
